# Validation of the Scale of Emotional Development-Short (SED-S) in Healthy Adults with an Intellectual Disability

**DOI:** 10.3390/jcm13175113

**Published:** 2024-08-28

**Authors:** Theresa Meinecke, Miriam Flachsmeyer, Tanja Sappok

**Affiliations:** 1 Center for Mental Health in Developmental Disabilities, Königin-Elisabeth-Herzberge Hospital, 13055 Berlin, Germany; theresa.meinecke@gmx.de; 2 Department of Child and Adolescent Psychiatry, Josefinum, 86154 Augsburg, Germany; flachsmeyer.miriam@josefinum.de; 3 Medical School and University Medical Center OWL, Mara Hospital, University Clinic for People with Neurodevelopmental Disorders, Bielefeld University, 33617 Bielefeld, Germany

**Keywords:** assessment, emotional development, intellectual disability, mental health, scale of emotional development-short

## Abstract

**Background:** The Scale of Emotional Development-Short (SED-S) assesses the level of emotional development (ED) of persons with intellectual disability (ID) in eight domains across five stages with reference ages from 0 to 12 years. The aim of this study was to apply and validate the SED-S in a sample of healthy adults with ID. **Method:** Eighty-three mentally healthy adults with ID were assessed using the SED-S. Factor analysis, Cronbach’s alpha, and correlational analyses were used to test the scale’s internal structure and associations. **Results:** The results showed that the eight-domain structure of the SED-S is supported by strong inter-domain correlations, a high Cronbach’s alpha, and a one-factor confirmatory factor analysis. The SED-S was associated with the severity of ID but not with age or gender. **Conclusions:** The SED-S can be used in non-clinical settings to better understand and meet the emotional needs of adults with ID.

## 1. Introduction

The prevalence rates of intellectual disability (ID) are estimated to be about 1% of the general population [1]. Persons with ID are especially vulnerable to experiencing mental health issues [2,3,4]. A recent meta-analysis suggested the prevalence of co-occurring mental disorders including severe challenging behaviors at 33.6% compared to 17% of mental disorders in the general population [3,5]. One of the most reported mental health problems is challenging behaviors including aggression, self-injury, property destruction, and stereotypies [2,6,7]. Consequences for persons with ID displaying challenging behaviors can be severe, including social exclusion, institutionalization, physical harm, abuse, misdiagnosis, and exposure to ineffective or aversive interventions [6]. Alarmingly, those exhibiting challenging behaviors are significantly more likely to receive psychotropic drugs, and prescription rates are higher than the diagnoses of mental disorders where such drugs are indicated [4]. Current guidelines, therefore, emphasize the need to distinguish between challenging behaviors, mental disorders, and developmentally appropriate behavior [8,9]. However, as the level of functioning decreases, symptoms start to lose their specificity, and mental disorders can take the form of unspecific behaviors that challenge both the persons and their environment [8,9,10]. Indeed, challenging behaviors can have many different causes, and every single behavior has to be carefully examined in every individual. The behaviors may derive from external factors, such as the loss of a significant individual or unexpected changes in the environment, or from internal factors, such as physical illnesses that cause pain, drug side effects, autism spectrum disorders, or from a combination of these environmental and person-associated factors. Some challenging behaviors may also be an inappropriate attempt to communicate or a maladaptive response in certain environments [10,11,12]. Thus, the interpretation of the behaviors against the background of the individual’s socio-emotional abilities becomes key, as these abilities are essential to the quality of relationships with the self and with other people and to the ability to cope with stress and to communicate and interact appropriately.

Therefore, to better understand the person-associated aspects of (challenging) behaviors, the developmental-dynamic approach was proposed [13,14]. It aims to acknowledge the socio-emotional brain functions, e.g., the ability to identify the emotional expressions of others, to properly interpret emotional cues and respond accordingly, and to self-soothe and regulate one’s own emotional outbursts and emotional expression [15]. The concept of emotional development (ED) describes the acquisition of emotional competencies following a progressive sequence of qualitative changes incorporating predominantly emotional but also social, sensorimotor, and cognitive functions against the background of normal development in infants [15]. The different components of ED are closely intertwined and result in an optimal adaptation to the environment. The maturation process is the product of multiple internal and environmental factors that reciprocally interact and stimulate the further development of the person. Being aware that development is a continuous process, for an assessment of the functional skills, a stage-wise model is supportive: The stepwise approach may guide the work in research and practice, as researchers and clinicians can agree upon a certain level of ED as assessed with a scientifically sound instrument.

Moreover, considering the level of ED could be supportive in determining which behaviors and needs are typical for a person in a certain emotional reference age group [16]. Each level of ED is associated with specific basic emotional needs and motivations, certain coping strategies, and adaptive behavior outcomes. Hermann et al. [17] described the behavioral phenomena for the different ED stages. In SED-S-1 (reference age 0–6 months), the behavior is characterized by simple motor actions (stereotypy, self-aggression, and hyperactivity) and seems to be detached from the environment (social withdrawal and isolation); in this developmental stage, the search for physical comfort predominates. In SED-S phase 2 (reference age 7–18 months), the challenging behavior is particularly evident as a high level of externalizing psychomotor activity and aggressiveness; the basic need in this phase is security. The third SED-S phase (19–36 months) is characterized by impulsive, defiant, and socially inappropriate behaviors and vocalizations; the search for autonomy becomes key in this level of ED. The behavioral peculiarities of SED-S phase 4 (reference age 4–7 years) encompass inappropriate speech, verbal self-regulation, and depressive-like symptoms such as mood swings, sadness, and reduced motivation. The individuals in this reference age group can name their own emotions, improve their affect regulation, and develop a Theory of Mind and an increasingly realistic view of the world. The behavioral phenomena can be viewed in terms of searching for identity. Recognizing the emotional needs of persons with ID early on may also contribute to the prevention of mental health issues, an aspect of mental health care that is often neglected [18].

The assessment of the level of ED thus requires a standardized and validated instrument. The Scale of Emotional Development–Short (SED-S) describes five levels of emotional development, which are aligned to the milestones of the developmental trajectories during childhood and the associated maturation of the respective brain circuits [19]. The five stages of ED are called adaptation (stage 1; emotional reference age: 0–6 months), socialization (stage 2; 7–18 months of age), first individuation (stage 3; 19–36 months of age), identification (stage 4; 37–84 months of age, or 4–7 years), and reality awareness (stage 5; 85–156 months of age, or 8–12 years).

The SED-S is a standardized interview consisting of 200 items in eight different domains describing observable behaviors typical for the five developmental stages. The SED-S has been validated in a sample of neurotypical children from age 0 to 12 years old [20]. An overall high agreement (81%) between the SED-S stage and the children’s chronological age was found. This indicates that the items describe behaviors that can be observed in typically developing children and that these behaviors are part of the trajectory of neurotypical development. Moreover, the inter-rater reliability on the domain level and internal consistency were high. Next, the SED-S has been studied in children with ID [21]. The authors reported moderate to strong correlations between the eight domains, high internal consistency, and a one-factor solution of the domains from a principal component analysis. In contrast to the neurotypical children, the level of ED was not significantly associated with the chronological age of the children with ID. This suggests that in the presence of ID, the process of ED may be delayed or incomplete [22]. In a study by Sappok, Heinrich, and Böhm (2020) in 327 adults with an ID and mental ill-health, the SED-S correlated negatively with the severity of ID (r_s_ = −0.654, *p* < 0.001) [23]. Flachsmeyer et al. (2023) applied the SED-S in a sample of 724 adults with ID, mostly with comorbid mental disorders and/or challenging behaviors from five study sites in three countries [24]. A confirmatory factor analysis showed a good fit-index for a one-factor model. The internal consistency assessed by Cronbach’s alpha was high (0.93). This study included 80 out of the 83 participants with ID without additional mental health problems of the current study. Despite some methodological overlaps with the study by Flachsmeyer et al. (2023), specifically, the CFA and the Cronbach’s alpha, the descriptive data (mean, median, standard deviation, and the distribution) of the stages of ED for each domain of the SED-S, the descriptives of the domain results for every severity of ID, the correlation analysis of every domain with the severity of ID, the inter-domain correlation matrix, and the correlation analysis with chronological ages have not yet been analyzed in healthy adults with an ID [24]. Moreover, the SED-S has been analyzed in different clinical contexts, e.g., the association with challenging behaviors or with autism spectrum disorder [17,23]. Autism spectrum disorder correlated with lower levels of emotional development (r_s_ = −0.316, *p* < 0.001) [23]. Similarly, the severity of challenging behaviors decreases in higher emotional reference age groups [17]. Additionally, this study describes the types of challenging behaviors displayed in the different reference age groups, e.g., emotional reference ages of 0–6 months were associated with social withdrawal, stereotypies, and aggression towards the self, while physical aggression was most prevalent in persons in the second phase of emotional functioning (7–18 months). In stage 3 (19–36 months), defiant and socially inappropriate behaviors could be observed. Persons with an emotional reference age of 4–7 years (SED-S stage 4) showed inappropriate speech, verbal self-regulation, and depressive-like behavioral aspects [17]. Based on an item analysis, Sappok et al. (2024) developed in a sample of 224 adults with an ID a brief version of the SED-S and validated this brief version of the SED-S in a second independent matched sample (*n* = 223) [25]. In this study, the item reliability ranged from 0.835 to 0.924 (Cronbach’s alpha). Overall, the results of the brief version agreed with the original SED-S in *p_O_* = 0.7. Another study compared the SED-S with other instruments assessing the level of ED, namely, the Scheme for Appraisal of Emotional Development (SAED), the Scale for Emotional Development—Second Revision (${SED-R}^{2}$) and the Schaal voor Emotionele Ontwikkeling—Lukas (SEO-Lukas) [26]. The SED-S results correlated most with the SEO-Lukas (Goodman and Kruskal gamma = 1; Cohen’s weighted kappa = 0.936) followed by the SAED (γ = 0.809; ð = 0.343) and least with the ${SED-R}^{2}$ (γ = 0.665; ð = 0.182). The stage of ED assessed with the SED-S was lower than the ED results measured with the SAED but higher than with the ${SED-R}^{2}$ and most similar to the SEO-Lukas. The Cronbach’s alphas were high, ranging from 0.853 to 0.975.

The aim of this study was to analyze and standardize the SED-S in adults with ID without comorbid mental disorders. The data of this paper were separately collected in sheltered living facilities in Berlin to report and describe the level of emotional functioning in adults with ID without psychiatric disorders. To that end, the current study provides the psychometric properties of the SED-S in a non-clinical sample of adults with ID. More specifically, we analyzed the construct validity and internal consistency of the eight domains of the SED-S and its associations with the severity of ID, age, and gender. We expected the eight domains of the SED-S to be internally consistent and best described as one construct and for there to be significant associations of the SED-S with the severity of ID but not with age and gender.

## 2. Materials and Methods

### 2.1. Design and Procedure

The study was conducted from 2017 to 2020 at the Berlin treatment Center for Mental Health in Developmental Disabilities, a psychiatric hospital in Berlin, Germany that specializes in the treatment of mental disorders in adults with an ID. Eighty-three adults with ID were recruited by contacting sheltered workplaces and living places in Berlin. The inclusion criteria were age > 18 years and a diagnosis of ID. The exclusion criteria were the presence of challenging behaviors, mental disorders, and/or autism spectrum disorders. The absence of mental disorders was confirmed by a careful review of the medical records and biographical information and an interview with the caregivers. The assessment of the SED-S was conducted by a clinical psychologist. Further data such as age, sex, somatic diseases, and living and working situation were assessed. The level of ID was measured with the Disability Assessment Schedule (DAS) [27].

### 2.2. Measures

#### 2.2.1. The Scale of Emotional Development-Short (SED-S)

The SED-S is a semi-structured interview to assess the level of ED in persons with ID [19]. It is conducted by trained professionals with one or more informants, i.e., a person who knows the individual with ID well, preferably from different environments (e.g., workplace, home). In the current study, one or more informants were present. The informants interviewed for the SED-S were most frequently close caregivers (70.3%, German: “Bezugsbetreuer”; a caregiver within an institution with a close professional relationship and responsibility for the person in need of care), followed by caregivers (16.9%), mothers (6%), others (3.6%), and, in one case, the father. Five items per domain and level of ED describe observable behaviors, resulting in a total of 200 items. Items are endorsed if they describe typical behaviors of the individual and scored dichotomously (yes/no). For example, an item in the domain Regulating Affect for SED-S stage 3 states: “Frustration finds expression in physical restlessness, temper tantrums and stubbornly oppositional behavior”. The items are answered with “yes” if the description is considered typical of the assessed person with ID. Per domain, the minimum score is zero, and the maximum score is five. The stage with the most ‘yes’ responses is the domain score. As such, every domain is rated from one to five, indicating the stage of ED. The overall or scale score is determined by ordering the eight domain scores and choosing the fourth-lowest level of ED. The minimum score for the overall scale is one, indicating stage 1, the maximum stage is five, indicating stage 5. The full domain names (in brackets) were abbreviated for the remaining article as follows: Body (Relating to his/her Own Body), Others (Relating to Significant Others), Object (Dealing with Change/Object Permanence), Emotion (Differentiating Emotions), Peers (Relating to Peers), Material (Engaging with the Material World), Communication (Communicating with Others), and Affect (Regulating Affect).

#### 2.2.2. Disability Assessment Schedule (DAS)

All participants were assessed with the DAS [27,28,29] to determine the severity of ID as recommended by the WHO [30]. The DAS is an informant-based structured interview that poses several questions about adaptive behaviors in four parts: continence, self-help skills, communication, and (cognitive) skills. More specifically, the domains include questions about toilet use (continence), eating, washing, and getting dressed (self-help skills); type of communication, comprehension, use of communication, and pronunciation (communication); and reading, writing, counting, and household abilities (cognitive skills). Depending on the availability or absence of these behaviors, points are awarded and summed to a total score (minimum 15 points; maximum 71). The total score is again categorized into the corresponding severity levels of ID (mild, moderate, severe, profound). It was found to be a reliable measure when conducted by trained professionals [27]. Evidence about its validity is provided by correlation analysis with the Colored Progressive Matrices and the Columbia Mental Maturity Scale [28].

### 2.3. Data Analysis

Data were analyzed in R [31]) and jamovi [32]. No missing data or outliers were detected. The sample size is sufficient to perform a CFA as the tested model was relatively simple (one factor, eight indicators) and, therefore, was potentially sufficient for the current analysis. Due to the ordinal nature of some of the variables and violations of normality and continuity, appropriate non-parametric estimation procedures were chosen. ID severity was ordered from mild to profound (mild, moderate, severe, profound), and ED was ordered from SED-S 1 to 5 (1, 2, 3, 4, 5). Some of the data (80/83 participants) have also been included in the large-scale validation study by Flachsmeyer et al. [24]. The focus of the paper by Flachsmeyer et al. (2023) was on the overall SED-S scores; neither an in-depth analysis on domain level nor an analysis of the differential levels of ED according to the severities of ID were applied [24].

#### 2.3.1. Descriptive Analyses

The sample characteristics were analyzed descriptively (mean, median, frequency, standard deviation). Further descriptive statistics of the SED-S on the scale and domain levels were calculated. Whether the average age differed significantly between the severity level of ID was tested using an ANOVA.

#### 2.3.2. Associations of the SED-S with the Severity of ID

The association of the SED-S and the severity of ID (DAS) was assessed with a correlation analysis. Spearman’s rank correlation coefficients were computed between the domains and the SED-S overall score. The strength of the correlations was interpreted as follows: strong (0.7–0.9), moderate (0.4–0.6), and weak (0.1–0.3) [33]. In a second step, the SED-S overall and domain results for every severity of ID were tested for significant differences with Kruskal–Wallis tests and subsequent pairwise comparisons. The results are presented in a bar graph. Spearman rho rank correlation coefficients between the SED-S, the domain scores, and the level of ID were computed. The overlap of the reference ages of the level of ED (assigned by the SED-S) and the level of ID (assigned by the DAS) were compared, and the percentages of the expected and discrepant stages of ED were calculated (see Sappok et al. [16,19] for reference ages). A Kruskal–Wallis test on the scale level was applied to test for age differences between the stages of ED. To test for gender differences on the SED-S overall score, a Mann–Whitney U test was used. 

Exploratorily, a previously applied definition of the evenness of developmental profiles based on Sappok, Heinrich, and Böhm [23] was used to calculate the percentage of even and uneven developmental profiles: “An SED-S profile was considered ‘even’ if at least five domains scored at the same [stage] or if at least three SED-S domains scored at the same [stage] and the other SED-S scores were at adjacent [stages] of ED”.

#### 2.3.3. Construct Validity

To evaluate the construct validity, a confirmatory factor analysis (CFA) [34] with eight indicators (domain scores) and one factor (ED) was calculated. We opted to test a one-factor model of the SED-S, since in previous research, a Principal Component Analysis supported a one-dimensional solution [21], and, theoretically, all the domains of the SED-S are thought to be part of a larger construct of emotional development. Moreover, Kline [35] suggested that when a one-factor model cannot be rejected, it makes little sense to start examining more complex factor models. Taken together, we considered the one-factor model the most appropriate one for testing the dimensionality of the SED-S. A diagonally weighted least squares (DWLS) estimation method was applied [35]. DWLS has been recommended for use with ordinal data [33,36,37]. The model fit was tested with the chi-square statistic (*p* > 0.05, non-significant) and further evaluated using some of the most widely used Goodness of Fit indicators with traditional cut off scores as recommended by Hu and Bentler [38]: the Comparative Fit Index (CFI > 0.95) and the Root Mean Square Error of Approximation (RMSEA < 0.06) and Standardized Root Mean Square Residual (SRMR < 0.08). The parameter estimates of the eight domain variables were calculated and compared with one another.

#### 2.3.4. Internal Consistency

Cronbach’s alpha was calculated to assess the internal consistency of the scale. Cronbach’s alpha assumes that all items (in our case, the domain scores) have equal covariances with the latent construct and equal factor loadings (tau-equivalence), and it has been widely criticized for being overinterpreted as the estimator of reliability [39,40,41]. However, since we used Cronbach’s alpha in conjunction with a test of dimensionality and correlational analyses, we considered its use appropriate, and it was informative to be able to compare it to previous studies on the SED-S [20,21]. A Cronbach’s alpha > 0.7 was rated as satisfactory [42,43].

In sum, the construct validity and internal consistency of the SED-S were considered supported if the eight domains were all significantly correlated, a one-factor model was upheld by CFA, and Cronbach’s alpha was satisfactory.

### 2.4. Ethics

The study was approved by the ethics committee of the initiating hospital in Berlin (approval 22 November 2016) and by the ethics commission of the Charité Universitätsmedizin Berlin (Ethics vote: EA2/193/16). The study was in line with the requirements of the 1975 Declaration of Helsinki. All participants or their legal guardians gave written informed consent for the participation. The informants gave their verbal consent before participating in the interview.

## 3. Results

### 3.1. Sample Description

The average age in this sample was 39.1 years, ranging from 19 to 76. More males than females were included (60.2%). The range of the severity of ID was mild (*n* = 17; 20.5%), moderate (*n* = 24; 28.9%), severe (*n* = 17; 20.5%), and profound (*n* = 25; 30.1%). The average age for those with mild ID was 38.1 (SD = 16.04), with moderate ID 38.83 (SD = 13.94), with severe ID 40.94 (SD = 13.34), and for those with profound ID 38.68 (SD = 12.01). These differences in age were not significant when tested in an ANOVA (F = 0.145, *p* = 0.933). Some common somatic comorbid diagnoses included movement disorders (42.2%), epilepsy (33.7%), visual impairments (13.3%), and hearing impairments (9.6%).

### 3.2. Correlations of the Level of ED and the Severity of ID

Correlational analyses between the SED-S and the severity of ID revealed highly significant (*p* < 0.001) negative associations for the overall score (r_s_ = −0.753). Lower stages of ED were associated with more severe forms of ID and vice versa. The severity of ID correlated with lower levels of ED. This pattern of association could also be detected at the domain level. However, it is noticeable that the domains Communication (r_s_ = −0.803), Body (r_s_ = −0.744), and Material (r_s_ = −0.743) showed strong correlations with ID (r_s_ > 0.7), whereas the domains Peers (r_s_ = −0.546), Affect (r_s_ = −0.552), Object (r_s_ = −0.595), and Others (r_s_ = −0.598) showed only moderate correlations (r_s_ < 0.6). All correlations were significant at the level of *p* < 0.001.

### 3.3. Association of the Level of ED Depending on the Severity of ID

Table 1 displays the association of the overall level of ED and the severity of ID. Those with mild ID tend to be categorized more often into higher stages of ED than those with more severe forms of ID. The bold figures indicate the expected SED-S stage based on the reference ages of the respective severity of ID. Overall, 62.65% of participants exhibited SED-S stages as expected by the severity of ID. The percentage of expected stages was lower in mild ID (11.76%) than in moderate (54.17%), severe (70.59%), and profound (100%) ID. This indicates that in higher levels of ID the emotional reference ages tend to be lower than expected from the intellectual reference ages.

The SED-S average overall score differed significantly depending on the severity of ID (χ^2^; = 51.02, df = 3, *p* < 0.001, ε^2^ = 0.622). Dwass–Steel–Critchlow–Fligner pairwise comparisons revealed differences in ED between mild and severe (W = −4.204, *p* = 0.019), mild and profound (W = −7.694, *p* < 0.001), moderate and severe (W = −3.96, *p* = 0.032), moderate and profound (W = −8.2, *p* < 0.001), and between profound and severe ID (W = −6.124, *p* < 0.001). Between mild and moderate ID, no significant difference in the level of ED was detected (W = −0.658, *p* = 1.00).

### 3.4. Association of the Level of ED Depending on the Severity of ID on Domain Level

**Figure 1:**
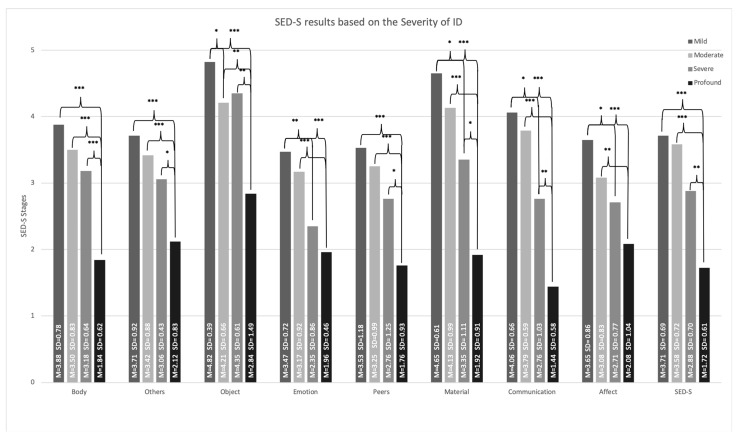
Mean values of the SED-S total score and its domains stratified by severity of ID. Note: columns display the level of ED for several severities of ID; * *p* < 0.05; ** *p* < 0.01; *** *p* < 0.001; *n* = 83. M = Mean; SD = Standard Deviation.

The means and standard deviations of the SED-S results according to the severity levels of ID for the overall results and on domain level are depicted in Figure 1. The brackets indicate significant differences in the level of emotional development between the different levels of ID.

The medians of the SED-S overall and domain results stratified by the severity of ID are displayed in Table 2. It further shows the mean and standard deviation of the SED-S results of the entire sample. 

### 3.5. Evenness of the SED-S Profile

The results of the evenness of the developmental profile analysis revealed that 66.27% of the participants had an “even” developmental profile. Of those, 34.94% met the criteria of having ≥ 5 domain scores at the same stage, and 60.24% had ≥ 3 equal domain scores.

### 3.6. The SED-S and Age

The mean age of the participants was 39.9 (SD = 16.1) in stage 1, 36.3 (SD = 10.5) in stage 2, 41.1 (SD = 15.6) in stage 3, 38.2 (SD = 11.9) in stage 4, and 41.8 (SD = 17.5) in stage 5. The average chronological age did not differ significantly depending on the SED-S overall results (χ^2^ = 1.45; df = 4, *p* = 0.836); the effect size measure was small (ε^2^ = 0.0176).

### 3.7. The SED-S and Gender

The average SED-S overall score for women was 2.76 (median = 3; SD 1.091; SE = 0.190; *n* = 33) and for men was 3.0 (median = 3; SD 1.5; SE = 0.148; *n* = 50). A Mann–Whitney U test revealed no differences between males and females on a scale level (U = 741; *p* = 0.420; r = 0.102).

### 3.8. Frequencies of Several SED-S Domain Scores

**Figure 2:**
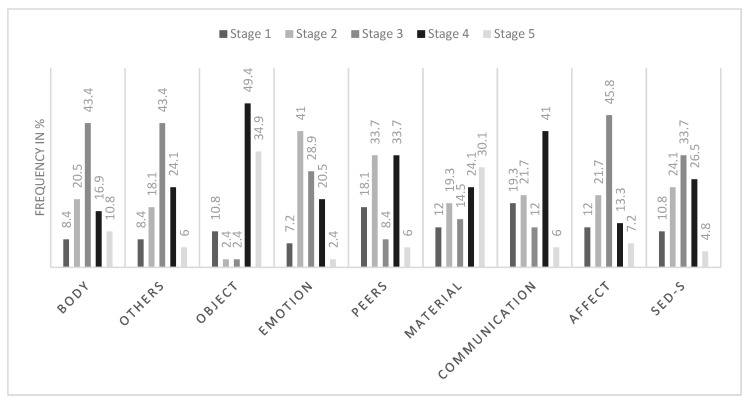
Distribution of the stages of ED. Note: Figure 2 depicts the frequency distribution (as a percentage) of the domain scores. All participants were assigned a stage of ED for each of the eight domains. Hence, every domain distribution amounts to 100%.

The frequency distribution of the stages on the domain and scale levels is depicted in Figure 2. The percentages are based on the 83 persons with ID included in the sample.

For the overall score of the SED-S, most participants (33.7%) were assigned to stage 3, followed by stages 4 (26.5%) and 2 (24.1%). The Body, Others, and Affect domains are similarly distributed as the overall score, while for the Object domain, most participants were categorized into stages 4 or 5. The distribution of the Emotions domain indicates a tendency towards lower levels of ED, with most participants in stage 2. The Peers domain shows two peaks—one in stage 2 and one in 4. In the Material domain, most participants were rated at stage 5, with decreasing frequency in the order of the declining stages. In the Communication domain, most participants were categorized into stage 4 followed by stage 1 or 2. The frequency distribution depending on the severity of ID can be found in the Supplementary Materials.

### 3.9. Construct Validity

**Figure 3:**
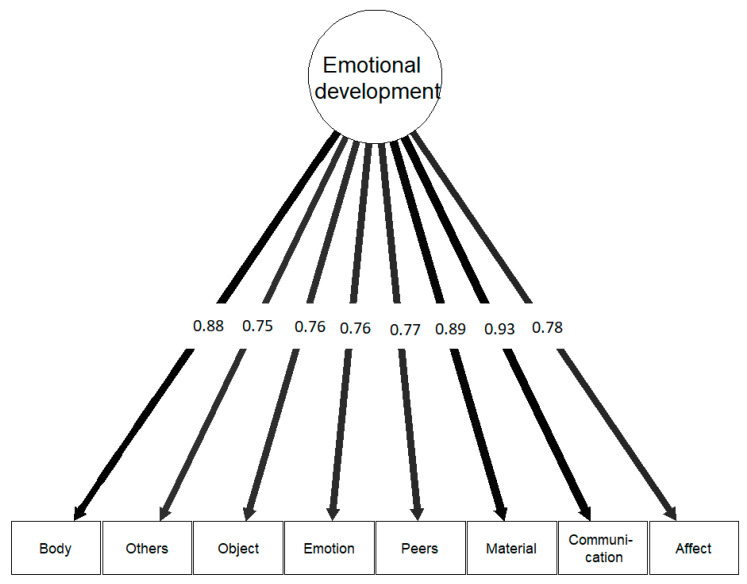
The one-factor model of the SED-S in healthy adults with ID. Note: Graphic representation of the one-factor model of the SED-S. Note: The model parameters are standardized. The width of the arrows depends on the strength of the factor loadings.

Figure 3 depicts the one-factorial model with the standardized parameter estimates of the eight domain scores.

The chi-square goodness of fit test of the one-factor CFA of the eight domains of the SED-S rendered a non-significant solution (χ^2^ = 8.388, df = 20, *p* = 0.989). The CFI was 1.000, the RMSEA was 0.000 (90% CI [0.000; 0.000]; *p* = 0.997), and the SRMR was 0.034. This means that the one-factor model of the SED-S is upheld. All eight domains can be significantly predicted by one underlying factor with standardized parameter estimates ranging from 0.750 for the Others domain to 0.934 for Communication. The domains ordered from the strongest to the weakest association were Communication, Material, Body, Affect, Emotion, Others, Peers, and Object. The same order was revealed for the variance explained. This implies that one underlying latent construct can account for much of the variance observed in the domain scores.

Table 3 shows the parameter estimates from the CFA for the eight domains of the SED-S. 

### 3.10. Domain Correlations and Cronbach’s Alpha

The Cronbach’s alpha of the eight-domain scores of the SED-S was 0.92 (CI [0.89; 0.94], suggesting high internal consistency of the eight domains in measuring ED. A correlation matrix of the eight domains and the overall score can be found in the Supplementary Materials. The domains showed correlations ranging from 0.409 to 0.759 (*p* < 0.001). The overall score also correlated significantly with the domain scores, correlating most strongly with the Communication domain (r_s_ = 0.858; *p* < 0.001) and least strongly with the Object domain (r_s_ = 0.618, *p* < 0.001). The moderate to high correlations between the eight domains suggest that they are all related and might measure aspects of the same underlying construct.

## 4. Discussion

This study provides an in-depth systematic description and evaluation of the SED-S in healthy adults with ID outside of a clinical setting. The level of ED can be assessed in male and female persons with ID across several ages. Studying the SED-S in a sample of adults with the diagnosis of ID-only offered the opportunity to assign the level of ED to the respective severity of ID. The results showed that all participants could be categorized into a stage of ED; however, the level of ED was often below the expected cognitive reference age groups. Especially in mild ID (88%) but also to a lesser degree in moderate (46%) and severe ID (29%), the emotional reference ages were lower than the cognitive reference ages as assigned by the severity of ID. Not a single person in this study had a level of ED that was higher than the expected cognitive reference age. Connecting the descriptive results of the SED-S in the current study with the underlying model of ED yields insights into what may constitute “normal” or “typical” levels of emotional behaviors, needs, and fears in adults with ID. This may serve as a basis for an understanding of what can be expected socially and emotionally from persons whose intellectual functioning is limited. As the level of ED may differ from the severity of ID, it should be evaluated in addition to IQ testing. Moreover, the results may provide basic information about the methods of interaction, differentiating emotions, and regulating affect that are typical for people in a certain level of ED to better differentiate typical behaviors from psychopathology [17]. Based on the respective level of emotional functioning as determined by the SED-S, individually tailored treatment and support can be offered, especially in the case of situations that are stressful for the person.

The SED-S was constructed to assess the level of ED in persons with ID based on the observations in eight different domains [19]. The results suggest that all these domains of ED are related and can best be explained by one underlying theoretical construct, which explained between 56% and 87% of the observed variance in the domain scores. The standardized factor loadings and the amount of variance explained indicated that a theoretical latent construct of ED is strongly associated with how someone communicates with others, engages in the material world, and relates to his or her own body (i.e., the domains Communication, Material, and Body), and to a lesser degree but still substantially with the other five domains. The correlational analyses revealed similar patterns. The constructed overall SED-S score is also strongly associated with the Communication, Material, and Body domains and is closely followed by moderate to strong correlations with the other five domains. A high Cronbach’s alpha supports the internal consistency of the eight domains in measuring ED.

Overall, these results are in line with previous validation studies of the SED-S in children [21] and a mostly clinical sample of adults with ID [24]. Moreover, 66.4% of participants had an even developmental profile, i.e., a small to moderate with-person variation between the domain scores. In summary, the analyses can be interpreted as supporting the construct validity and internal consistency of the SED-S. Despite the general findings that all eight domains are associated with each other, a closer look at the results shows that some variation remains unexplained by an overall latent construct or the overall score. Differences in the domains are also reflected in the distributions and their average scores. This could be interpreted as every domain adding unique information to the construct of ED and measuring separate aspects of the same underlying construct. Interestingly, the Object domain showed the lowest explained variance and correlated less strongly with the overall score of the SED-S. A reason for that could be that object permanence may be either present or absent. A trend towards this dichotomy is reflected in the distribution of the stages of the Object domain, where most participants were either categorized into stage 2 or stage 4. Sterkenburg et al. [21] also found the Object domain to be least associated with the other domains. They suggested that object permanence might be more of a cognitive ability and thereby less strongly associated with other aspects of ED. Moreover, some items of the Object domain have not been found to be age specific [20]. A similar pattern of the Object domain was also found in the study by Flachsmeyer et al. [24]. Therefore, deleting this domain from the scale may be an option.

The analysis of the evenness of profiles showed that at least one-third of the participants had an uneven developmental profile. Differences between the domains are possible. Those differences may lead to an over- or underestimation of the ED when looking only at the overall score of the SED-S. This could be particularly important when considering the individual and his or her emotional needs, regulation strategies, and ways of interacting with peers and caregivers. Therefore, not only the overall level of ED but also the developmental profile should be acknowledged, particularly in those with an uneven profile. Especially in persons with uneven profiles, using additional instruments focusing on certain aspects of development, such as the Reflective Functioning Questionnaire—Mild to Borderline intellectual Disabilities (RFQ-MBID) for the assessment of mentalizing abilities or instruments to assess the attachment style, may be indicated [44].

As this study found no significant correlations between the SED-S results and the chronological age, the age of people with ID may not adequately predict their level of ED. It stresses that the assessment of ED is important throughout every age because development is possible and should also be encouraged in adulthood. The absence of gender differences on the SED-S showed that the level of ED can be measured in men and women with ID. The absence of an association with chronological age and gender supports the divergent validity of the SED-S.

The significant negative association between the SED-S and ID indicates that less severe forms of ID show higher developmental stages of the SED-S and vice versa. The results of the SED-S on the domain and the overall level revealed a similar pattern of changes in ED levels depending on the severity of ID. These findings are in line with previous research [21,23]. This can be considered to support the convergent validity of the SED-S since intellectual and emotional functions are expected to be parallel under ideal conditions [13,14]. Interestingly, the Communication, Material, and Body domains showed the strongest correlations with the severity of ID, while the Emotion, Others, Object, Affect, and Peers domains were correlated only moderately. This could in part be explained by similar underlying mechanisms between emotional and intellectual development. For example, some aspects of ED, such as naming feelings, require a certain level of language mastery. Some degree of construct overlap between the Communication and Body domains with the measure of the severity of ID (DAS) could have further influenced the results. Nevertheless, the finding that the Emotion, Others, Object, Affect, and Peers domains correlate only moderately with ID severity suggests that it is especially important to assess these aspects in addition to intellectual functioning. Further analyses on the domain level of the SED-S and its association with ID severity are needed to better understand the nature of how these aspects emerge and change in adults with ID.

The analyses of the expected and discrepant stages of ED according to the suggested reference ages in the ICD-10 and the SED-S manual showed that delays in ED are common, and ED tends to be lower than the level of ID. This underlines the importance of the additional assessment of the level of ED, along with an assessment of the severity of ID.

The current study was subject to some limitations. One limitation of this study was the size of the sample. This is pilot study examining the level of emotional functioning in healthy adults with ID for the first time. Further studies are warranted to evaluate the emotional reference ages and the associated adaptive behaviors in more detail. The sample presented in this study was recruited from Germany. Further research is needed to examine the emotional reference ages in other countries as well. The sample size is relatively small to perform a CFA. The discussion about the sample size requirements for conducting a CFA is inconclusive, and many aspects, such as the number of factors, indicators, and estimation methods, influence the sample size [36,45]. The model tested here was relatively simple (one factor, eight indicators) and, therefore, was potentially sufficient for the current analysis. Factors that decrease the required sample size are simple models, high reliability scores (Cronbach’s alpha here is 0.92), no missing data, and a higher number of indicators per factor (8) [46]. These factors all apply to the current study. Moreover, Wolf et al. (2013) conducted a Monte Carlo simulation study with different specified models varying the number of factors, indicators, and observed factor loadings [45]. According to the Monte Carlo simulation, the one-factor model of the eight domain indicators with observed factor loadings between 0.65 and 0.8 would require a sample size of 50 or less. Lastly, a CFA was conducted on a much larger sample (*n* > 500) with very similar results for the parameter estimates as well as the model fit parameters. Therefore, we believe that the current sample size is sufficient to apply a CFA to estimate the factorial model of the SED-S.

Regarding the construct validity of the SED-S, another limitation is that we tested only a one-factor model instead of testing alternative models against each other. Our rationale was to test the simplest model first, and we had evidence from previous research for a one-factor solution [21]. Nevertheless, future research on the internal structure of the SED-S could consider looking beyond unidimensional models of the construct of ED and perhaps consider alternative models of ED that might allow for dynamic changes between different aspects of ED, such as network models. In further studies, the emotional reference ages may be assessed in people with borderline intellectual functions. Within these, the expanded version of the scale including stage 6 (reference ages 13–17 years old) should be applied. In further studies, the model should be tested according to the severity of ID.

The dependence on the domain scores in evaluating the validity of the SED-S represents a drawback of the current study. Methodological difficulties on the item level, such as the construct overlap between the domains and/or difficulties in differentiating between the five stages of ED, may have influenced the current results and should be considered in future studies of the SED-S.

The use of the alpha coefficient could be seen as a limitation of the study, as it is based on stringent assumptions of unidimensionality and tau-equivalence [43] and there have been critical discussions regarding its usefulness [40,47]. However, given the evidence of unidimensionality in the current study by the CFA and the additional examination of the correlations between the domains, we concluded that reporting the alpha coefficient was informative and useful as it made it possible to compare it to previous studies on the SED-S. Future research should consider alternative reliability estimations and investigate the test-retest and inter-rater reliability.

The assessment of the severity of ID with the screening tool DAS may limit the study to some extent. The DAS includes four questions about communication, which may constitute a certain degree of construct overlap with the SED-S Communication domain and might have influenced the strength of the association. Moreover, the DAS measures the severity of ID based on daily living skills instead of a structured cognitive assessment.

Finally, other well-established developmental theories exist, such as the attachment theory, the self-determination theory, and the social information processing model. More work needs to be performed to develop and test assessments and interventions based on these theories that are valid and effective in populations of people with ID. This may further expand our understanding of the inner processes and dynamics that lead to a person with a neurodevelopmental disorder displaying a certain behavior.

## 5. Conclusions

This study signifies a systematic description of the SED-S in adults with ID outside of a clinical setting. It suggests that in the absence of mental disorders and ASD, adults with ID also display delays in emotional brain functions as shown by the lower levels of ED. The SED-S showed high internal consistency, and a one-factorial model of ED was supported, suggesting that the SED-S is a consistent and unidimensional measure of ED. The use of an overall score is supported; nevertheless, the high proportion of uneven profiles (35%) suggests the need to assess emotional functioning on the domain level. The level of ED may be below the reference ages expected from the severity of ID; therefore, an assessment of ED may be beneficial, especially in people with unexpected behaviors. No association with age and gender was found. Overall, this study is a further component in a comprehensive validation process of the SED-S and its use in non-clinical environments. An understanding and recognition of the level of ED could help caregivers to determine whether certain behaviors are appropriate for the respective person and thereby contribute to supporting the mental health and quality of life in this highly vulnerable population of adults with ID.

## Figures and Tables

**Table 1:** Expected SED-S stages based on the severity of ID.

	Frequencies *n* (%)					
Level of ID	Stage 1 (0–0.5 Years)	Stage 2 (0.5–1.5 Years)	Stage 3 (1.5–3 Years)	Stage 4 (4–7 Years)	Stage 5 (8–12 Years)	Total
Mild (9–12 years)	0	0	7 (41)	8 (47)	**2 (12)**	17 (100)
Moderate (6–9 years)	0	1 (4)	10 (42)	**11 (46)**	**2 (8)**	24 (100)
Severe (3–6 years)	0	5 (29)	**9 (52)**	**3 (18)**	0	17 (100)
Profound (0–3 years)	**9 (36)**	**14 (56)**	**2 (8)**	0	0	25 (100)

Note: This table shows the counts and frequencies in percentage of the stages of ED (SED-S overall score) by the severity of ID. Numbers printed in bold signify an expected stage of ED based on reference ages.

**Table 2:** SED-S total and domain results.

	Severity of ID (*n*)	SED-S	Body	Others	Object	Emotion	Peers	Material	Communication	Affect
Median	all (83)	3	3	3	4	3	2	4	3	3
	mild (17)	4	4	4	5	3	4	5	4	4
	moderate (24)	4	3	3.5	4	3	4	4	4	3
	severe (17)	3	3	3	4	2	2	3	2	3
	profound (25)	2	2	2	4	2	2	2	1	2
Mean	all (83)	2.90	3.01	3.01	3.95	2.70	2.76	3.41	2.93	2.82
SD	all (83)	1.070	1.080	1.010	1.210	0.959	1.260	1.410	1.290	1.050

**Table 3:** Parameter estimates of the CFA.

ED =~	Estimate	Standardized Estimate	Variance Explained	Std. Error	z-Value	*p*-Value	Variance
Body	1.000	0.879	0.773				0.227
Others	0.853	0.750	0.563	0.054	15.894	<0.001	0.437
Object	0.860	0.756	0.572	0.070	12.238	<0.001	0.428
Emotion	0.866	0.762	0.581	0.067	12.891	<0.001	0.420
Peers	0.874	0.769	0.591	0.067	13.044	<0.001	0.409
Material	1.010	0.888	0.789	0.058	17.385	<0.001	0.212
Communication	1.062	0.934	0.872	0.050	21.116	<0.001	0.128
Affect	0.888	0.781	0.610	0.068	13.117	<0.001	0.390

Note: The numbers are based on the R analysis using the DWLS estimation method. The table shows the estimate, the standardized estimate, the z-value and *p*-value fo the test of significance for the paramenter estimates, and the variance. The variance explained was added by taking the squared standardized estimate.

## Data Availability

Data are available on request.

## Supplementary Materials

The following supporting information can be downloaded at: https://www.mdpi.com/article/10.3390/jcm13175113/s1, Table S1: Frequency Distribution (%) of the Stages of ED for the entire sample and stratified by severity of ID; Table S2: Domain Correlation Matrix.

## References

[1] Maulik P.K., Mascarenhas M.N., Mathers C.D., Dua T., Saxena S. (2011). Prevalence of intellectual disability: A meta-analysis of population-based studies. Res. Dev. Disabil..

[2] Cooper S.-A., Smiley E., Morrison J., Williamson A., Allan L. (2007). Mental ill-health in adults with intellectual disabilities: Prevalence and associated factors. Br. J. Psychiatry J. Ment. Sci..

[3] Mazza M.G., Rossetti A., Crespi G., Clerici M. (2020). Prevalence of co-occurring psychiatric disorders in adults and adolescents with intellectual disability: A systematic review and meta-analysis. J. Appl. Res. Intellect. Disabil..

[4] Sheehan R., Hassiotis A., Walters K., Osborn D., Strydom A., Horsfall L. (2015). Mental illness, challenging behaviour, and psychotropic drug prescribing in people with intellectual disability: UK population based cohort study. BMJ.

[5] Steel Z., Marnane C., Iranpour C., Chey T., Jackson J.W., Patel V., Silove D. (2014). The global prevalence of common mental disorders: A systematic review and meta-analysis 1980–2013. Int. J. Epidemiol..

[6] O’Brien M.J., Matson J.L. (2020). Challenging behaviors and dual diagnosis. Handbook of Dual Diagnosis: Assessment and Treatment in Persons with Intellectual Disorders.

[7] Emerson E., Kiernan C., Alborz A., Reeves D., Mason H., Swarbrick R., Mason L., Hatton C. (2001). The prevalence of challenging behaviours: A total population study. Res. Dev. Disabil..

[8] (2015). NICE Guideline. Challenging Behaviour and Learning Disabilities: Prevention and Interventions for People with Learning Disabilities Whose Behaviour Challenges. https://www.nice.org.uk/guidance/ng11.

[9] Gardner W., Došen A., Griffiths D.M., King T. (2006). Practice Guidelines for Diagnostic, Treatment and Related Support Services for People with Developmental Disabilities and Serious Behavioral Problems.

[10] Deb S., Perera B., Krysta K., Ozer M., Bertelli M., Novell R., Wieland J., Sappok T. (2021). The European guideline on the assessment and diagnosis of psychiatric disorders in adults with intellectual disabilities. Eur. J. Psychiatry.

[11] Došen A., De Groef J. (2015). What is normal behaviour in persons with developmental disabilities?. Adv. Ment. Health Intellect. Disabil..

[12] Royal College of Psychiatrists (2007). Challenging Behaviour: A Unified Approach.

[13] Došen A. (2005). Applying the developmental perspective in the psychiatric assessment and diagnosis of persons with intellectual disability: Part I—Assessment. J. Intellect. Disabil. Res..

[14] Došen A. (2005). Applying the developmental perspective in the psychiatric assessment and diagnosis of persons with intellectual disability: Part II—Diagnosis. J. Intellect. Disabil. Res..

[15] Sappok T., Hassiotis A., Bertelli M., Dziobek I., Sterkenburg P. (2022). Developmental Delays in Socio-Emotional Brain Functions in Persons with an Intellectual Disability: Impact on Treatment and Support. Int. J. Environ. Res. Public Health.

[16] Sappok T., Zepperitz S., Hudson M. (2021). Meeting Emotional Needs in Intellectual Disability: The Developmental Approach.

[17] Hermann H., Berndt N., Lytochkin A., Sappok T. (2022). Behavioural phenomena in persons with an intellectual developmental disorder according to the level of emotional development. J. Intellect. Disabil. Res..

[18] Allen D., Langthorne P., Tonge B., Emerson E., McGill P., Fletcher R., Došen A., Kennedy C. (2013). Towards the prevention of behavioural and psychiatric disorders in people with intellectual disabilities. J. Appl. Res. Intellect. Disabil..

[19] Sappok T., Barrett B.F., Vandevelde S., Heinrich M., Poppe L., Sterkenburg P., Vonk J., Kolb J., Claes C., Bergmann T. (2016). Scale of Emotional Development—Short. Res. Dev. Disabil..

[20] Sappok T., Böhm J., Birkner J., Roth G., Heinrich M. (2019). How is your mind-set? Proof of concept for the measurement of the level of emotional development. PLoS ONE.

[21] Sterkenburg P.S., Kempelmann G.E.M., Hentrich J., Vonk J., Zaal S., Erlewein R., Hudson M. (2021). Scale of emotional development–short: Reliability and validity in two samples of children with an intellectual disability. Res. Dev. Disabil..

[22] Hodapp R., Zigler E., Cicchetti D., Cohen D. (1995). Past, present and future issues in the developmental approach to mental retardation and developmental disabilities. Developmental Psychopathology.

[23] Sappok T., Heinrich M., Böhm J. (2020). The impact of emotional development in people with autism spectrum disorder and intellectual developmental disability. J. Intellect. Disabil. Res..

[24] Flachsmeyer M., Sterkenburg P., Barrett B., Zaal S., Vonk J., Morisse F., Gaese F., Heinrich M., Sappok T. (2023). Scale of Emotional Development—Short: Reliability and validity in adults with intellectual disability. J. Intellect. Disabil. Res..

[25] Sappok T., Barrett B., Lutter S. (2024). A brief version of the Scale of Emotional Development—Short. J. Intellect. Disabil. Res..

[26] Sappok T., Morisse F., Flachsmeyer M., Vandevelde S., Ilic M., Barrett B.F. (2023). Brief report comparing the Scale of Emotional Development—Short (SED-S) with other scales for emotional development. J. Intellect. Disabil. Res..

[27] Holmes N., Shah A., Wing L. (1982). The Disability Assessment Schedule: A brief screening device for use with the mentally retarded. Psychol. Med..

[28] Meins W., Süßmann D. (1993). Evaluation of an adaptive behaviour classification for mentally retarded adults. Soc. Psychiatry Psychiatr. Epidemiol..

[29] Auwetter J. (1998). Psychopharmakoprävalenz bei Geistig Behinderten Erwachsenen: Ein Vergleich zwischen Verschiedenen Wohnformen. Ph.D. Thesis.

[30] World Health Organization (1996). Division of Mental Health. ICD-10 Guide for Mental Retardation. WHO/MNH/96.3. WHO IRIS. https://apps.who.int/iris/handle/10665/63000.

[31] R Core Team (2020). R: A Language and Environment for Statistical Computing.

[32] The Jamovi Project (2021). Jamovi (Version 1.6). https://www.jamovi.org.

[33] Koğar H., Yilmaz Koğar E. (2015). Comparison of Different Estimation Methods for Categorical and Ordinal Data in Confirmatory Factor Analysis. Eğitimde Psikolojide Ölçme Değerlendirme Değerlendirme.

[34] Jöreskog K.G. (1969). A general approach to confirmatory maximum likelihood factor analysis. Psychometrika.

[35] Kline R.B. (2011). Principles and Practice of Structural Equation Modeling.

[36] Li C.H. (2016). Confirmatory factor analysis with ordinal data: Comparing robust maximum likelihood and diagonally weighted least squares. Behav. Res..

[37] Holgado-Tello F.P., Morata-Ramírez M.Á., Barbero-García M.I. (2018). Confirmatory factor analysis of ordinal variables: A simulation study comparing the main estimation methods. Av. Psicol. Latinoam..

[38] Hu L., Bentler P.M. (1999). Cutoff criteria for fit indexes in covariance structure analysis: Conventional criteria versus new alternatives. Struct. Equ. Model. Multidiscip. J..

[39] Sijtsma K. (2009). On the use, the misuse, and the very limited usefulness of Cronbach’s Alpha. Psychometrika.

[40] McNeish D. (2018). Thanks coefficient alpha, we’ll take it from here. Psychol. Methods.

[41] Raykov T., Marcoulides G.A. (2019). Thanks coefficient alpha, we still need you!. Educ. Psychol. Meas..

[42] Lance C.E., Butts M.M., Michels L.C. (2006). The sources of four commonly reported cutoff criteria: What did they really say?. Organ. Res. Methods.

[43] Revelle W., Condon D.M. (2019). Reliability from α to ω: A tutorial. Psychol. Assess..

[44] Derks S.D.M., Willemen A.M., Vrijmoeth C., Sterkenburg P.S. (2023). Lessons learned from the adaptation of the Reflective Functioning Questionnaire (RFQ) for Dutch people with mild to borderline intellectual disabilities. PLoS ONE.

[45] Wolf E.J., Harrington K.M., Clark S.L., Miller M.W. (2013). Sample size requirements for Structural Equation Models: An evaluation of power, bias, and solution propriety. Educ. Psychol. Meas..

[46] Kyriazos T. (2018). Applied Psychometrics: Sample Size and Sample Power Considerations in Factor Analysis (EFA, CFA) and SEM in General. Psychology.

[47] Akoglu H. (2018). User’s guide to correlation coefficients. Turk. J. Emerg. Med..

