# Supplementary material for: Validation of the Scale of Emotional Development-Short (SED-S) in Healthy Adults with an Intellectual Disability
Source: J Clin Med. 2024 Aug 28;13(17):5113. doi: 10.3390/jcm13175113 (PMC11395714; doi:10.3390/jcm13175113)
Supplement: Supplementary file 1 [file jcm-13-05113-s001.zip › jcm-3122151-supplementary.pdf]

Supplementary Materials

Table S1: *Frequency Distribution (%) of the Stages of ED for the entire sample and stratified by severity of ID.*

	Severity of ID	Stage 1	Stage 2	Stage 3	Stage 4	Stage 5
SED-S	all	11	24	34	27	5
	mild	0	0	41	47	12
	moderate	0	4	42	46	8
	severe	0	29	52	18	0
	profound	36	56	8	0	0
Body	all	8	21	43	17	11
	mild	0	0	35	41	24
	moderate	0	4	58	21	17
	severe	0	6	76	12	6
	profound	28	60	12	0	0
Others	all	8	18	43	24	6
	mild	0	12	24	47	18
	moderate	0	17	33	42	8
	severe	0	6	82	12	0
	profound	28	32	40	0	0
Object	all	11	2	2	49	35
	mild	0	0	0	18	82
	moderate	0	4	0	67	29
	severe	0	0	6	53	41
	profound	36	4	4	52	4
Emotion	all	7	41	29	21	2
	mild	0	6	47	41	6
	moderate	0	29	29	38	4
	severe	18	35	41	6	0
	profound	12	80	8	0	0
Peers	all	18	34	8	34	6
	mild	6	18	12	47	18
	moderate	0	33	13	50	4
	severe	12	47	0	35	6
	profound	48	36	8	8	0
Material	all	12	19	15	24	30
	mild	0	0	6	24	71
	moderate	0	13	4	42	42
	severe	0	29	24	29	18
	profound	40	32	24	4	0
Communication	all	19	22	12	41	6
	mild	0	0	18	59	24
	moderate	0	4	17	75	4
	severe	6	47	12	35	0
	profound	60	36	4	0	0
Affect	all	12	22	46	13	7
	mild	0	6	41	35	18
	moderate	0	21	58	13	8
	severe	6	29	53	12	0
	profound	36	28	32	0	4

Table S2: *Domain Correlation Matrix.*

Variables								
Others	0.617							
Object	0.564	0.484						
Emotion	0.566	0.587	0.476					
Peers	0.528	0.451	0.409	0.551				
Material	0.759	0.551	0.561	0.620	0.633			
Communication	0.737	0.622	0.556	0.702	0.662	0.725		
Affect	0.601	0.495	0.520	0.544	0.571	0.590	0.671	
SED-S	0.808	0.729	0.618	0.766	0.674	0.807	0.858	0.715
	Body	Others	Object	Emotion	Peers	Material	Communication	Affect

Note: This table shows the inter-domain correlations of the SED-S and with the overall score. All correlations are positive and significant at level $p < .001$.
